# To: Complementary of modified NUTRIC score with or without C-reactive protein and subjective global assessment in predicting mortality in critically ill patients

**DOI:** 10.5935/0103-507X.20200100

**Published:** 2020

**Authors:** Dino Moretti, Nicolás Sebastián Rocchetti

**Affiliations:** 1 Intensive Care Unit, Hospital “Eva Perón” - Santa Fe, Argentina.

**To the Editor**

We have read the article “Complementarity of modified NUTRIC score with or without C-reactive protein and subjective global assessment in predicting mortality in critically ill patients” by Oliveira et al.^(1)^ with great interest. The authors found excellent agreement between the modified Nutrition Risk in the Critically Ill (NUTRIC) score and the NUTRIC with C-reactive protein (CRP) score; in addition, the combination of NUTRIC score and subjective global assessment was a good predictor of increased risk of death at 28 days. These findings led them to suggest that using an inflammatory biomarker, such as CRP, to assess and stratify the nutritional risk of critical patients in the intensive care unit (ICU) may not be necessary. However, we highlight certain points of disagreement with the authors.

First, the authors used a different categorization of CRP within the NUTRIC scoring system, stratifying CRP levels into tertiles for analysis (< 68, 68 to 167 and ≥167mg/dL). This approach is welcome, since their investigation is the second study that uses this biomarker; however, the severity of the study population was relatively low, based on low average Acute Physiology, Age and Chronic Health Evaluation II (APACHE II) and Sequential Organ Failure Assessment (SOFA) scores, a 60% mechanical ventilation rate and an average ICU stay of 8 days. Therefore, their results are not necessarily generally applicable given the severity of patients in other ICUs, such as the ICU at our center, where NUTRIC with CRP was originally studied;^(2)^ further valuation studies are merited.

Second, CRP has become a widely used biomarker that can be monitored at virtually any center with an ICU. We can thus investigate its usefulness for the diagnosis, management and prognosis of multiple pathologies.^(3)^ In critically ill patients, malnutrition is closely related to the underlying inflammatory state, and depleted body protein is a central consideration.^(4)^ Within a solid and coherent physiopathological framework for reasoning about nutrition in such patients, dispensing with a widely available inflammatory biomarker (CRP) that has been validated for enhancing the value of the NUTRIC score would not be reasonable, particularly given that Oliveira et al.^(1)^ found that a higher risk of death at 28 days was better predicted using NUTRIC-CRP alone (hazard ratio - HR = 2.685; 95% confidence interval - 95%CI 1.423 - 5.064; p = 0.002) or in combination with malnutrition (HR = 4.112; 95%CI 1.738 - 9.727) than if CRP were not utilized (for mNUTRIC alone: HR = 1.827; 95%CI 1.029 - 3.244; p = 0.040; for mNUTRIC with malnutrition: HR = 2.167; 95%CI 1.029 - 4.563).

It remains to be clarified whether the observation of a higher percentage of patients classified as high risk by NUTRIC-CRP than by mNUTRIC (34% *versus* 28%) is replicated in subsequent studies, which could suggest different nutritional therapeutic behaviors for patients classified as low risk by mNUTRIC and high risk by NUTRIC-CRP.

In short, we must be cautious when making recommendations about dispensing with a widely available biomarker (such as CRP) when using NUTRIC. However, we agree with the message conveyed by the authors regarding the complementarity between the NUTRIC score, regardless of which NUTRIC approach is used, and subjective global assessment.
